# Delayed sleep phase syndrome in adolescents: prevalence and correlates in a large population based study

**DOI:** 10.1186/1471-2458-13-1163

**Published:** 2013-12-11

**Authors:** Børge Sivertsen, Ståle Pallesen, Kjell Morten Stormark, Tormod Bøe, Astri J Lundervold, Mari Hysing

**Affiliations:** 1 Division of Mental Health, Norwegian Institute of Public Health, Kalfarveien 31, 5018, Bergen, Norway; 2 Uni Health, Uni Research, Bergen, Norway; 3 Department of Psychiatry, Helse Fonna HF, Haugesund, Norway; 4 Department of Psychosocial Science, University of Bergen, Bergen, Norway; 5 Norwegian Competence Center for Sleep Disorders, Haukeland University Hospital, Bergen, Norway; 6 The Regional Centre for Child and Youth Mental Health and Child Welfare, Uni Health, Uni Research, Bergen, Norway; 7 Department of Clinical Psychology, University of Bergen, Bergen, Norway; 8 K.G. Jebsen Centre for Research on Neuropsychiatric Disorders, University of Bergen, Bergen, Norway; 9 Department of Biological and Medical Psychology, University of Bergen, Bergen, Norway

**Keywords:** Delayes sleep phase syndrome, Sleep, Prevalence, Correlates, Epidemiology

## Abstract

**Background:**

The aims of this study were to estimate the prevalence of Delayed Sleep Phase Syndrome (DSPS) in adolescence, and to examine the association to insomnia and school non-attendance.

**Methods:**

Data stem from a large population based study in Hordaland County in Norway conducted in 2012, the ung@hordaland study. In all, 10,220 adolescents aged 16–18 years (54% girls) provided self-reported data on a range of sleep parameters: DSPS was defined according to the International Classification of Sleep Disorders, Revised (ICSD-R) criteria, while insomnia was defined according to the Quantitative Criteria for Insomnia. Other sleep parameters included time in bed, sleep duration, sleep efficiency, oversleeping, sleep onset latency, wake after sleep onset, subjective sleep need, sleep deficiency, sleepiness and tiredness. Sleep data were calculated separately for weekdays and weekends. Data on school non-attendance were provided by official registers.

**Results:**

The prevalence of DSPS was 3.3%, and significantly higher among girls (3.7%) than boys (2.7%). There was a strong overlap between DSPS and insomnia, with more than half of the adolescents with DSPS also meeting the criteria for insomnia (53.8% for boys and 57.1% for girls). Adolescents with DSPS had significantly higher odds ratios (OR) of non-attendance at school. After adjusting for sociodeographical factors, insomnia and depression, the adjusted ORs for days of non-attendance were OR = 3.22 (95% CI: 1.94-5.34) for boys and OR = 1.87 (95% CI: 1.25-2.80) for girls. A similar effect was found for hours of non-attendance for boys, with an adjusted OR = 3.05 (95% CI: 1.89-4.92). The effect for girls was no longer significant after full adjustment (OR =1.48 [95% CI: 0.94-2.32]).

**Conclusions:**

This is one of the first studies to estimate the prevalence of DSPS in adolescents. The high prevalence of DSPS, and overlap with insomnia, in combination with the odds of school non-attendance, suggest that a broad and thorough clinical approach is warranted when adolescents present with symptoms of DSPS.

## Background

During adolescence a general pattern of delayed bedtimes but early risetime due to school obligations renders the sleep duration shorter than both the adolescents themselves and experts deem necessary [1]. The delayed sleep phase is a consequence of a biological delay in the circadian rhythm [2] and a slower build up of sleep pressure that occurs during puberty [3]. Also, social factors, such as reduced parental influence of the adolescents’ sleep patterns, may contribute to this developmental pattern [4]. However, when given the opportunity, the adolescents’ sleep will often be of normal length and in accordance with the individually perceived sleep need [1]. While a delayed sleep onset and rebound sleep during weekends seem to reflect normal developmental patterns occurring frequently in adolescence, for some the mismatch between sleep pattern and social obligations, accompanied by daytime impairment, will be at a level that meets the diagnostic criteria for Delayed Sleep Phase Syndrome (DSPS).

To obtain a diagnosis of DSPS according to the newly published 5^th^ edition of the Diagnostic and Statistical Manual of Mental Disorders (DSM-V), there are three criteria which needs to be met: 1) the characteristic misalignment of sleep, in conjunction with 2) excessive sleepiness or insomnia and 3) significant daytime impairment in social, occupational or other important areas of functioning. According to the DSM-V, the use of sleep diaries or actigraphy is also required. Although, no studies have used these new criteria, two previous studies, surveying the general population with age ranges from adolescence to adults, based their estimates on multi-phased assessments with screening questionnaires, sleep diaries as well as on clinical interviews. These two studies concluded on prevalence rates from 0.13% to 0.17% [5,6]. Although the rate of DSPS peaks during adolescence, with a suggested prevalence of over 7% [7], the exact prevalence rate remains uncertain. Previous epidemiological studies of adolescents using different operationalizations of DSPS have estimated prevalence rates from 1.9% [8] in a study basing the definition of DSPS on the International Classification of Sleep Disorders (ICSD-R), to 8.4% [9] employing a much wider definition. In a 2011 review of studies on sleep characteristics and sleep disorders in adolescents published from 1999 to 2010, the limited number of studies did not allow for an estimate of DSPS in this age group [10]. However, the review concluded that DSPS was likely to be underestimated in adolescence due to the general sleep pattern in adolescents mirroring some of the sleep characteristic of DSPS [10]. Also since trend studies suggest an increase in sleep onset difficulties among adolescents [11] new and updated surveys of the prevalence of DSPS are warranted.

Potential gender differences in DSPS have rarely been reported in the literature, probably due to lack of statistical power caused by small sample sizes. One exception was a British study of adolescents from 1988 who found a male predominance of 10:1 [12]. In contrast, a Norwegian epidemiological study using a less stringent operationalization of DSPS recently found no evidence for gender differences in Delayed Sleep Phase [13]. On a general basis adolescent boys seem to have a somewhat more delayed sleep phase than adolescent girls [14].

Insomnia, a sleep disorder which is characterized by difficulties initiating and maintaining sleep and related daytime impairments, is another prevalent sleep disorder during adolescence with prevalence rates between 14-24% depending on diagnostic criteria [1]. When co-occurring at a diagnostic level and the insomnia is occurring parallel with the DSPS, the DSPS takes precedence over the insomnia diagnosis according to ICSD-2 [15]. In the DSM-V, insomnia is rated as one of the sleep related functional impairments of DSPS in addition to sleepiness, and it is stated as one of the important differential diagnostic, but also co-occurring conditions [7]. However, the rate of co-occurrence of these conditions has received little focus in the literature. In one of the few studies focusing on DSPS and insomnia, Johnson et al. [8], using definition of DSPS according to the ICSD-R, found that DSPS did *not* account for a significant proportion of insomnia cases as defined by the DSM-IV.

The functional impact on occupational or other important areas is one of the diagnostic criteria of DSPS. This is in line with the research status on sleep problems in general as a major risk factor for both short- and long-term sick leave [16,17], and permanent work disability [18-22], which causes soaring economical consequences for the society [23-25]. To the best of our knowledge, no previous studies have investigated such consequences among individuals with DSPS, neither among adolescents nor adults. However, being characterized by circadian misalignment, short sleep duration and accompanying impaired daytime functioning, DSPS has been associated with poor academic performance [9,13], as well as lower school attendance [10]. Having an internal clock misaligned with school hours, it is likely that adolescents with DSPS also are at higher risk of increased school absence, which again may be a pathway to later school dropout. The overlap between DSPS and other co-occurring conditions such as depression and insomnia may account for some of the functional impairment. Elevated symptoms of depression have been demonstrated in this group compared to good sleepers [13,26]. Still, larger studies using well-defined operationalizations of both DSPS and depression are lacking, and thus the unique associations between DSPS and functional impairment remain unknown.

Based on these considerations, the aims of the current study were: 1) to estimate the prevalence and sleep characteristic of DSPS in a general population sample of adolescents; 2) to examine the overlap between DSPS and insomnia; 3) to examine the association between DSPS and non-attendance at school, and if this could be accounted for by co-occurring depression and insomnia; and 4) to investigate if there are gender differences in the association between DSPS and non-attendance at school.

## Methods

### Procedure

In this population-based study, we used data from the ung@hordaland-survey of adolescents in the county of Hordaland in Western Norway. All adolescents born between 1993 and 1995 and all students attending secondary education during spring 2012 were invited. Data were collected during spring 2012. Adolescents in secondary education received information per e-mail, and time during regular school hours was allocated for them to complete the questionnaire. A teacher was present to organize the data collection and to ensure confidentiality. Those not in school received information by postal mail to their home addresses. Survey staff was available on a phone number for both the adolescents and school personnel for answering queries. The study was approved by the Regional Committee for Medical and Health Research Ethics in Western Norway. A previous population-based study from the Hordaland county, the Hordaland Health Study (HUSK), found the county to be representative for the Norwegian population [27].

### Sample

A total of 19,439 adolescents were invited to participate in the survey, of whom 10,220 agreed, yielding a participation rate of 53%. Sleep variables were checked for validity of answers based on preliminary data analysis, resulting in data from 374 adolescents being omitted due to obvious invalid responses (e.g. negative sleep duration and sleep efficiency). 508 adolescents did not complete all items needed to define DSPS. Thus, the total sample size in the current study was 9,338. There were no significant age differences between participants being excluded due to invalid/missing response on the sleep items, but there were more boys (61.8%) than girls (38.2%) among the excluded participants.

### Instruments

#### Socio-demographical and clinical information

All participants indicated their vocational status, with response options being “high school student”, “vocational training” or “not in school”. Maternal and paternal education were reported separately with three response options; “primary school”, “secondary school”, “college or university”. Parental cohabitant situation was assessed using a dichotomized variable (yes/no) asking if the parents currently live together. Family economy was assessed by asking the participants how their family economy is compared to most others. Response alternatives were 1 = “approx. like most others”; 2=”better economy”, and 3=”poorer economy”. Smoking was assessed by asking the participants if they smoke. Response options included 1=”yes, daily”, 2=” yes, sometimes, but not daily”, 3=”no, not now, but previously”, 4=”no, not anymore”, and 5=”no”. Current smoking was operationalized as endorsing the two first response alternatives, whereas choosing response alternatives 3–5 was coded as non-current smoker. Alcohol use was assessed by asking if they had ever tried alcohol (yes/no). Body-mass index (BMI) was calculated from self-reported body weight (kilogram) divided by squared height (meter^2^).

#### Delayed sleep phase syndrome (DSPS)

The following questions were used to operationalize DSPS: “At what time do you usually go to bed”, “How much time does it take before you fall asleep (hours and minutes)”, “When do you usually get out of bed in the morning”, “How many nights per week do you have difficulties falling asleep (0–7)”, “How many nights per week do you have problems with nightly awakenings (0–7)”, “How often do you oversleep (“never”, “seldom”, “sometimes”, “mostly”, “always”)”. The participants provided sleep data separately for weekdays and weekends. No information regarding the time-frame of these symptom was available. To approximate assessment of the ICSD-R criteria for DSPS, the following criteria were used (based on the aforementioned sleep items), as specified in Johnson et al. published in Pediatrics [8] 1) minimum 1-hour shift in sleep-onset AND wake times from the weekdays to the weekend, 2) complaint of frequent (≥ 3 days per week) difficulty falling asleep, 3) report of little or no (≤ 1 day per week) difficulty maintaining sleep, and 4) frequent difficulty awakening (oversleep “sometimes” or more often).

#### Insomnia

Difficulties initiating and maintaining sleep (DIMS) were rated on a three point Likert-scale with response options “not true”, “somewhat true” and “certainly true”. Given a positive response (“somewhat true” or “certainly true”), participants were then asked how many days per week they experienced problems either initiating or maintaining sleep. Duration of DIMS was rated in weeks (up to three weeks) months (up to 12 months) and a last category over a year. A joint question on tiredness/sleepiness was rated on a three point Likert-scale with response options “not true”, “somewhat true” and “certainly true”. If confirmed (“somewhat true” or “certainly true”) participants reported the number of days per week they experienced sleepiness and tiredness, respectively. Insomnia was operationalized according to Lichstein et al.’s Quantitative Criteria for Insomnia [28]: self-reported DIMS at least three times a week, with a duration of six months or more, in addition to reporting SOL and/or WASO of more than 30 minutes, as well as tiredness or sleepiness at least three days per week.

#### Other sleep variables

Self-reported bedtime and rise time were indicated in hours and minutes using a scroll down menu with five minutes intervals and were reported separately for weekends and weekdays. Time in bed (TIB) was calculated by subtracting bedtime from rise time. Sleep onset latency (SOL) and wake after sleep onset (WASO) were indicated in hours and minutes using a scroll down menu with five minutes intervals, and sleep duration was defined as TIB minus (SOL + WASO). Sleep efficiency was calculated as sleep duration divided by TIB multiplied by 100 (reported as percentage). Subjective sleep need was reported in hours and minutes, and sleep deficiency was calculated separately for weekends and weekdays, subtracting total sleep duration from subjective sleep need.

#### Depression

Symptoms of depression were assessed using a short version of the Mood and Feelings Questionnaire (SMFQ) [29]. The SMFQ comprises 13 items assessing depressive symptoms rated on a 3-point Likert scale (“correct”, “sometimes correct” and “not correct”). A high internal consistency between the items and a strong uni-dimensionality have been shown in previous population-based studies [30], and was recently confirmed in a study including adolescents from the same survey as the one used in the present study [31]. For purposes of the present study, depression was defined as a score above the 90^th^ percentile of the Total SMFQ-score.

#### School non-attendance

Non-attendance at school was assessed using both register-based and self-reported information. Official register-based data on non-attendance were provided by Hordaland County Council, and days and hours of absence during the last semester (6 months) were included. For the purpose of the present study, we used both the mean of these two items, as well as dichotomous variables using the 90^th^ percentile as the cutoff (≥2 days and ≥5 hours, respectively). Although not the main outcome variables in the current study, we also assessed self-reported non-attendance at school, using the following two items: “How many days of non-attendance have you had the last month?” and “How many single hours (in addition to whole days) of non-attendance have you had the last month?” The two self-report variables were also used both continuously and dichotomously using the 90th percentile as the cutoff (≥3 days and ≥6 hours, respectively).

### Statistics

IBM SPSS Statistics 21 for Mac (SPSS Inc., Chicago, Ill) was used for all analyses. Independent sample t-tests and χ^2^-tests were used to examine differences in demographical, clinical and sleep variables in adolescents with and without DSPS. Between-group effect sizes (pooled SD) were calculated using the Cohen d formula [32]. Logistic regression analyses were used to assess the effect of DSPS on school non-attendance, both crude and adjusting for the following covariates included separately: age and parental education, insomnia, and depression. Finally, we examined a fully adjusted model in which all covariates were entered simultaneously. The logistic regression analyses were conducted separately for boys and girls.

## Results

### Demographical and clinical characteristics of the sample

The mean age of the 9,338 included adolescents was 17.0 years (range 16–19), and the sample included more girls (53.5%) than boys (46.5%). The vast majority (98%) were high school students.

Table 1 shows that the prevalence of DSPS was significantly higher among girls (3.7%) than boys (2.7%), yielding an overall prevalence rate of 3.3% (n = 306). The prevalence of DSPS increased with age: whereas 2.8% of adolescents aged 17 fulfilled the criteria for DSPS, the prevalence rates among 18- and 19-year olds were 3.1 and 4.2%, respectively (**χ**^2^(2) = 8.53, p = .014). There were no significant difference between the DSPS group and non-DSPS group in terms of vocational situation and maternal education, but there was a significant difference in parental education, with a higher percentage of the DSPS grouping having fathers with less education. The DSPS group also reported more depressive symptoms, with an SMFQ total score of 8.8 compared to 5.7 in the non-DSPS group (*P* < .001; Table 1).

**Table 1:** Demographical and clinical characteristics in adolescents with and without delayed sleep phase syndrome (DSPS) in the ung@hordaland study (n = 9,338)

	**No DSPS (n = 9032)**		**DSPS (n = 306)**			
	**%/mean**	**(SD)**	**%/mean**	**(SD)**	***P *- level**	**Cohen’s *d***
Age, mean	17.8	(0.8)	18.0	(0.8)	.006	0.25
Girls, %	96.3%		3.7%		.007	
Boys, %	97.3%		2.7%			
Vocational situation, %					.153	
In school	98.1%		99.3%			
Trainee	1.3%		0.0%			
Not in school	0.7%		0.7%			
Maternal education, %					.119	
University/College	48.7%		51.4%			
High school	41.4%		35.7%			
Primary school	9.9%		12.9%			
Paternal education, %					< .001	
University/college	43.1%		51.1%			
High school	46.7%		33.5%			
Primary school	10.3%		15.5%			
Parents live together, %					< .001	
No	32.0%		46.3%			
Family economy					.006	
Approx. like most others	67.8%		61.1%			
Better economy	25.2%		27.7%			
Poorer economy	6.9%		11.2%			
Current smoker	31.9%		35.9%		.225	
Ever tried alcohol	73.6%		87.6%		< .001	
Body mass index	22.2	3.6	22.2	3.5	.818	0.00
Insomnia, %	12.3%	(32.8)	55.9%	(49.7)	< .001	1.04
Depression (total SMFQ score)	5.7	(5.7)	8.8	(5.9)	< .001	0.53
Non-attendance, mean						
Self-reported days*	1.4	(2.4)	2.3	(2.6)	< .001	0.36
Self-reported hours*	2.1	(3.2)	3.7	(3.9)	< .001	0.45
Number of days^$^	3.9	(4.9)	7.1	(6.0)	< .001	0.58
Number of hours^$^	7.2	(10.8)	15.4	(14.5)	< .001	0.64

*Last month.

^$^Official register-based during last 6 months.

Table 2 shows sleep characteristics in the DSPS vs. non-DSPS group. Both bedtime and rise-time was significantly later in the DSPS group compared to the non-DSPS group, both during weekdays and weekends. Sleep duration (during weekdays), SOL, sleep efficiency, and the difference between weekdays and weekends were significantly poorer in the DSPS group, whereas there was no difference in WASO between the two groups. The DSPS group also reported a sleep deficiency of 3:38 hours during weekdays, but a *negative* sleep deficiency of 40 minutes during the weekends, a trend that was not found in the non-DSPS group. In addition, the DSPS group reported more tiredness and sleepiness during daytime, (all *Ps* < .001; see Table 2 for details).

**Table 2:** Sleep characteristics in adolescents with and without delayed sleep phase syndrome (DSPS) in the ung@hordaland study (n = 9,338)

	**No DSPS (n = 9032)**		**DSPS (n = 306)**			
	**Mean**	**SD**	**Mean**	**SD**	***P *- level**	**Cohen’s *d***
Weekdays						
Bedtime	23:17	00:59	23:46	01:04	< .001	0.42
Risetime	06:47	00:40	07:00	00:48	< .001	0.30
Time in bed	07:30	01:00	07:14	01:07	< .001	−0.20
Sleep duration	06:27	01:38	05:41	01:21	< .001	−0.45
Sleep efficiency (%)	84.8%	18.21	78.0%	13.2	< .001	−0.43
Oversleeping (“most days” or “always”)	5.0%	21.8	27.6%	44.8	< .001	0.64
Weekends						
Bedtime	01:31	01:29	02:07	01:16	< .001	0.35
Risetime	11:13	01:31	12:09	01:17	< .001	0.61
Time in bed	09:41	01:23	10:01	01:06	< .001	0.28
Sleep duration	08:38	01:52	08:28	01:05	.168	−0.07
Week days/weekends						
Sleep onset latency	00:47	00:57	01:21	00:50	< .001	0.62
Wake after sleep onset	00:14	00:38	00:11	00:23	.130	−0.16
Subjective sleep need	08:34	01:52	09:09	01:58	< .001	0.25
Sleep deficiency (weekdays)	02:07	02:29	03:28	02:22	< .001	−0.48
Sleep deficiency (weekends)	00:04	02:30	−00:40	02:12	< .001	−0.25
Sleepiness (days per week)	2.25	2.05	3.09	2.21	< .001	0.39
Tiredness (days per week)	3.68	1.86	4.87	1.80	< .001	0.65
Differences between weekdays and weekends						
Bedtime	02:27	02:17	02:21	00:59	.434	−0.09
Risetime	04:26	01:36	05:28	01:23	< .001	0.42
Sleep duration	02:11	01:33	02:47	01:25	< .001	0.38

### Overlap between DSPS and insomnia

**Figure 1:**
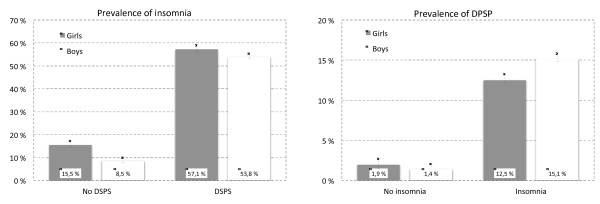
**Overlap between insomnia and delayed sleep phase syndrome (DSPS) among boys and girls in the ung@hordaland study (n = 9846).** Error bars represent 95% confidence intervals.

**Figure 2:**
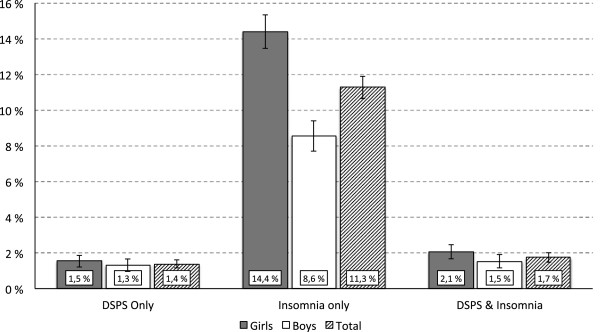
**Prevalence of adolescents with only delayed sleep phase syndrome (DSPS), only insomnia and both insomnia and DSPS among boys and girls in the ung@hordaland study (n = 9846).** Error bars represent 95% confidence intervals.

As detailed in Figures 1 and 2, there was a substantial overlap between DSPS and insomnia. The prevalence of insomnia was 53.8% for boys and 57.1% for girls in the DSPS group, compared to 8.5% and 15.56% in the non-DSPS group. Similarly, the prevalence of DSPS among insomniacs was 12.5% in girls and 15.1% in boys, compared to 1.9% and 1.4% in the non-insomnia group. The prevalence of DSPS only (without insomnia) was 1.4%, whereas 11.3% had insomnia without DSPS and 1.7% had both DSPS and insomnia.

### DSPS and school non-attendance

DSPS was significantly associated with school non-attendance, as detailed in Tables 1 and 3. Using official register-based non-attendance over last 6 months as the outcome variable, adolescents with DSPS reported a mean of 7.1 days and 15.4 hours of non-attendance, compared to 3.9 days and 7.2 hours in the non-DSPS group (all *Ps* < .001; both effect-sizes: 0.6). Boys had slightly higher crude OR of days of non-attendance compared to girls (OR = 4.6 vs. OR = 3.2), but there was no significant interaction effect between gender and DSPS on days of non-attendance (Wald = 1.67, df = 1, *P* = .196). A somewhat stronger gender difference was found for hours of non-attendance, with a significant interaction effect between gender and DSPS (Wald = 6.07, df = 1, *P* = .014). Adjusting for socio-demographics, depression, and especially insomnia, reduced the associations, but the associations between DSPS and school non-attendance remained significant in the fully adjusted models for both gender and outcomes, with the exception of hours of non-attendance among girls. No significant age differences were found for the associations between DSPS and school non-attendance.

**Table 3:** Multivariate logistic regressions analysis of delayed sleep phase syndrome (DSPS) as a risk factor for register-based school non-attendance among boys and girls in the ung@hordaland study (n = 9,338)

	**Days of non-attendance**^**$**^				**Hours of non-attendance**^**$**^			
	**Girls**		**Boys**		**Girls**		**Boys**	
**Adjustment variables**	**OR**	**95% CI**	**OR**	**95% CI**	**OR**	**95% CI**	**OR**	**95% CI**
Crude (DSPS)	3.15	2.22–4.70	4.57	2.93–7.14	2.37	1.60–3.53	4.90	3.23–7.45
Age, paternal education, cohabitant status and family economy	2.56	1.76–3.74	3.87	2.42–6.20	2.10	1.39–3.18	4.22	2.70–6.59
Ever tried alcohol	3.12	2.17–4.48	4.63	2.96–7.24	2.26	1.49–3.41	5.05	3.32–7.69
Depression	2.87	2.00–4.13	4.44	2.84–6.95	2.08	1.38–3.16	4.88	3.20–7.44
Insomnia	2.25	1.56–3.24	3.45	2.14–5.55	1.70	1.12–2.56	3.25	2.08–5.09
Fully adjusted model*	1.87	1.25–2.80	3.22	1.94–5.34	1.48	0.94–2.32	3.05	1.89–4.92

^**$**^Dichotomized at the 90th percentile.

*Adjusted for age, paternal education, cohabitant status, family economy, alcohol, insomnia and depression.

Additional analyses using self-reported school non-attendance during the last month as the outcome variable were also conducted. These findings showed the same pattern of associations and with similar gender differences. The crude ORs for self-reported days of school non-attendance were 1.86 (95%CI 1.33-2.60) for girls and 4.46 (95% CI 2.97-6.71) for boys . The corresponding odds for hours of school non-attendance were 2.75 (95% CI 1.91-3.98) for girls and 3.64 (95% CI 2.28-5.81) for boys. The associations were only slightly attenuated and remained significant in the fully adjusted analyses (results not shown).

## Discussion

The estimated prevalence of DSPS in the present population-based study was 3.3%. The overlap between insomnia and DSPS was high, with half of the adolescents reaching the criteria for DSPS also presenting with symptoms according to the insomnia criteria. Adolescents with DSPS had a higher rate of school absence than their peers, with an independent contribution of DSPS after adjusting for socio-demographical factors, depression and insomnia. Girls had a higher prevalence of DSPS than boys, but the level of school absence was higher in boys with DSPS.

The current prevalence rate of DSPS is comparable to the study by Johnson et al. from 2006 [8]. While both being community based studies, using similar operationalization, the adolescents in the present study were somewhat older, with an age spanning from 16 to 19 years compared to 13 to 16 years in the Johnson et al.’s study, which may contribute to explain the slightly higher prevalence rates in the current study. Not surprising, the current estimate is lower than reported in a previous Norwegian study from 2012 by Saxvig et al. (8.8%) which used less stringent criteria of delayed sleep phase [13]. However, when restricting the definition in the abovementioned study to only include those with oversleeping two or more times per week, the prevalence was reduced to 4.9%, which is more in accordance with the rate reported in the present study.

The sleep pattern among adolescents with DSPS exhibited the expected sleep characteristics, with shorter sleep duration due to later bedtime and early awakenings during the weekdays, as well as rebound sleep during the weekends, while their WASO was not significantly different from that of their peers. Adolescents with DSPS also had lower sleep efficiency and higher sleep deficiency compared to peers without DSPS. The present study confirmed that the sleep pattern of adolescents with DSPS is located at an extreme end of a continuum of normal sleep. According to Gradisar et al. [10], it may be difficult to distinguish characteristics of DSPS from sleep patterns that are normal during adolescence, and that one therefore runs the risk of underestimating the prevalence of DSPS. However, just representing the extreme of a continuum may also lead to an overestimation of the diagnosis: Whereas the diagnostic criteria remain the same across the life span, the sleep characteristics fluctuate depending on the age. The misalignment criteria of the DSPS is at a level that might be regarded as the norm for adolescents, with the observed mean difference in bedtime and risetime for weekdays versus weekends being 2:15 hours and 4:28 hours respectively [1]. What perhaps differentiate these normal sleep patterns from a diagnostic level of DSPS may therefore only partly be the sleep characteristics; more important are their functional impact and outcomes.

In accordance with the study by Johnson et al. [8], the present study defined oversleeping as one of the criteria of DSPS. Other functional sleep related factors such as tiredness and sleepiness was significantly higher in the DSPS group, but also prevalent in adolescents in general. The rate of frequent oversleeping was, however, not as prevalent, which in turn impacts the prevalence rate in the current study. This is also an example of how operationalization in epidemiological studies will impact the prevalence rates. We chose to use an operationalization with specific criteria that have been used previously. The new DSM-V diagnostic criteria on sleep wake disorder have chosen to use broad criteria to increase reliability in clinical diagnosis, making specific operationalizations for use in research more challenging. The current results still can inform on key aspects of the DSM-V diagnosis, including the importance of both insomnia as a co-occurring condition, and the functional impact on school attendance.

There was a significant association between DSPS and insomnia in the present sample, with half of the adolescents with DSPS also meeting the criteria for insomnia, as defined according to Lichstein’s Quantitative Criteria [28]. The conclusion by Johnson et al. [8] that insomnia does not account for a significant proportion of DSPS was supported by the current study, as most of the adolescents with insomnia did not have DSPS. Thus, insomnia warrants attention as a public health concern in its own right also in adolescence, and should not be viewed as merely a byproduct of DSPS. While insomnia is frequent in adolescents with DSPS, and thus in line as one of the functional impairment criteria in DSM-V (and as such can be regarded as a symptom of DSPS), many proportion of adolescents with DSPS do *not* have insomnia, and the impact of having one or both of the conditions is still not settled. It may be that while the misaligned sleep schedule temporarily precedes the insomnia, the problems with maintaining sleep may be accompanied by worry and catastrophic thoughts that may exacerbate the sleep problems and warrant clinical attention in its own right.

Few population-based studies on adolescents have assessed potential gender differences in DSPS, most likely due to the low overall prevalence rates and the need of large samples to detect statistically significant gender differences. In the previous epidemiological study by Johnson et al. [8], no gender specific rates were reported. The female preponderance in the present study was surprising given the higher male ratio in the study by Thorpy et al. [12], although a more even gender balance was found in a broader delayed sleep phase study from Norway [13]. Methodological differences may account for some of these differences. While girls had a higher rate of DSPS, the gender differences were not as marked as for insomnia, where there was an even higher female preponderance [1].

Adolescents with DSPS had a higher rate of non-attendance at school than their peers, emphasizing the functional impact of DSPS and mirroring the functional impact of sleep problems in adults. While girls had a higher rate of DSPS, the present study found a considerable higher odds of school absence in boys compared to girls. These results could illuminate some of the previously inconsistent gender prevalence rates. If the functional impact is higher among boys, as measured by an important outcome measure (such as school absence), this could instigate worry and health-seeking behavior among parents and teachers, and thus a diagnosis of DSPS would be more likely. This should lead to future research questions related to differences in characteristics of those who have the diagnosis of DSPS and those who meet all the diagnostic criteria except the functional impairment. It would also be of interest to examine if the same relations exist across other functional impairments, such as social relations and use of health care services. How school absence may impact academic performance remains unknown, although this may be one pathway linking DSPS to lower self-reported school grades found among adolescents with delayed sleep phase found in a previous study [13].

There are some methodological limitations of the present study that should be noted. First, the cross-sectional nature of the study does not allow for causal inferences. Thus, longitudinal studies concerning the relationship between pre-pubertal sleep patterns and later development of DSPS are needed to shed light on the developmental patterns. Secondly, tthe definition of DSPS represents another important limitation of the present study as it is based solely on self-report, and consequently lacks clinical evaluation and measurement by actigraphy or sleep diary. However, this is rarely possible in large epidemiological studies. Furthermore, while we did assess depression and insomnia, which accounted for some of the functional impact of DSPS in the present study, there may be other covariates not addressed in the current study that may explain parts of this association. For example, sleep phase misalignment may be a marker of more serious psychiatric disorders, which again may be related to school absence, such as a prodromal phases for more severe psychiatric disorders (e.g., bipolar disorder or schizophrenia). Third, depression was assessed by a self-report instrument, the SMFQ. As no validated cut-off exists for Norwegian adolescents, the 90th percentile on the total SFMQ score was chosen as an operationalization of depression. Clearly, this does not imply existence of a clinical diagnosis, such as MDD, and the lack of clinical interview in confirming a clinical diagnosis of depression is a limitation of the present study. In relation to this it should be noted that the SMFQ neither contains any sleep items nor items that assess any other vegetative symptoms. In contrast to conventional depression rating scales which normally contain such items, this prevents circularity and make the interpretation of associations between symptoms of sleep and affective problems unambiguous in the present study. Tiredness was included in the SMFQ, but the association to several sleep parameters was not higher for this item than for other depressive symptoms. Another limitation comprises the inclusion of a relatively low number of adolescents not in school. Although the few adolescents not attending school in the present study did not have a higher rate of DSPS, a higher participation rate among those adolescents would be needed to draw conclusions regarding this group specifically. Finally, the attrition from the study could affect generalizability, with a response rate of about 53% and with adolescents in schools overrepresented. Based on previous research from the former waves of the Bergen Child Study, non-participants often have more psychological problems than participants [33], and it is therefore likely that prevalences of both DSPS, insomnia and depression may be underestimated in the current study.

### Clinical implications

The high degree of overlap between DSPS and other conditions, such as depression and insomnia, warrants a thorough diagnostic evaluation and differential diagnosis when adolescents present with DSPS symptoms. While DSPS may preclude a diagnosis of insomnia, the symptomatic presentation may impact treatment choices.

The total score on the SMFQ in adolescents with DSPS in the current study (8.8) was comparable to the pre-treatment depression score in a RCT including adolescents with DSPS (total score SMFQ 7.5) [34]. In that study, the treatments effect of a combined cognitive behavioral therapy and bright light therapy showed a large reduction of depression symptoms, suggesting that both the sleep problems and the co-occurring depressive symptoms may be targeted through the same interventions [34].

## Conclusions

The epidemiology of DSPS is still in its infancy, and while the present study contributes to an understanding of the prevalence and correlates of the symptoms, more studies are needed to bridge the gap between our knowledge based on well-characterized DSPS in clinical trials, and knowledge from epidemiological studies. Future research should seek to examine if the functional impairment found in the current study is related to later educational outcomes and dropout from work life, which would indicate major individual and public health consequences.

## Competing interests

The authors declare that they have no competing interests.

## Authors’ contributions

Author KMS, AJL, BS, TB and MH were involved in acquisition of data. KM obtained funding. Authors BS and MH were responsible for conception and design of the study, conducted the statistical analysis and drafted the manuscript. Authors SP, KMS, TB and AJL gave critical revision of the manuscript for important intellectual content. Authors BS and MH had full access to all the data in the study and takes responsibility for the integrity of the data and the accuracy of the data analysis. All authors read and approved the final manuscript.

## Pre-publication history

The pre-publication history for this paper can be accessed here:

http://www.biomedcentral.com/1471-2458/13/1163/prepub
